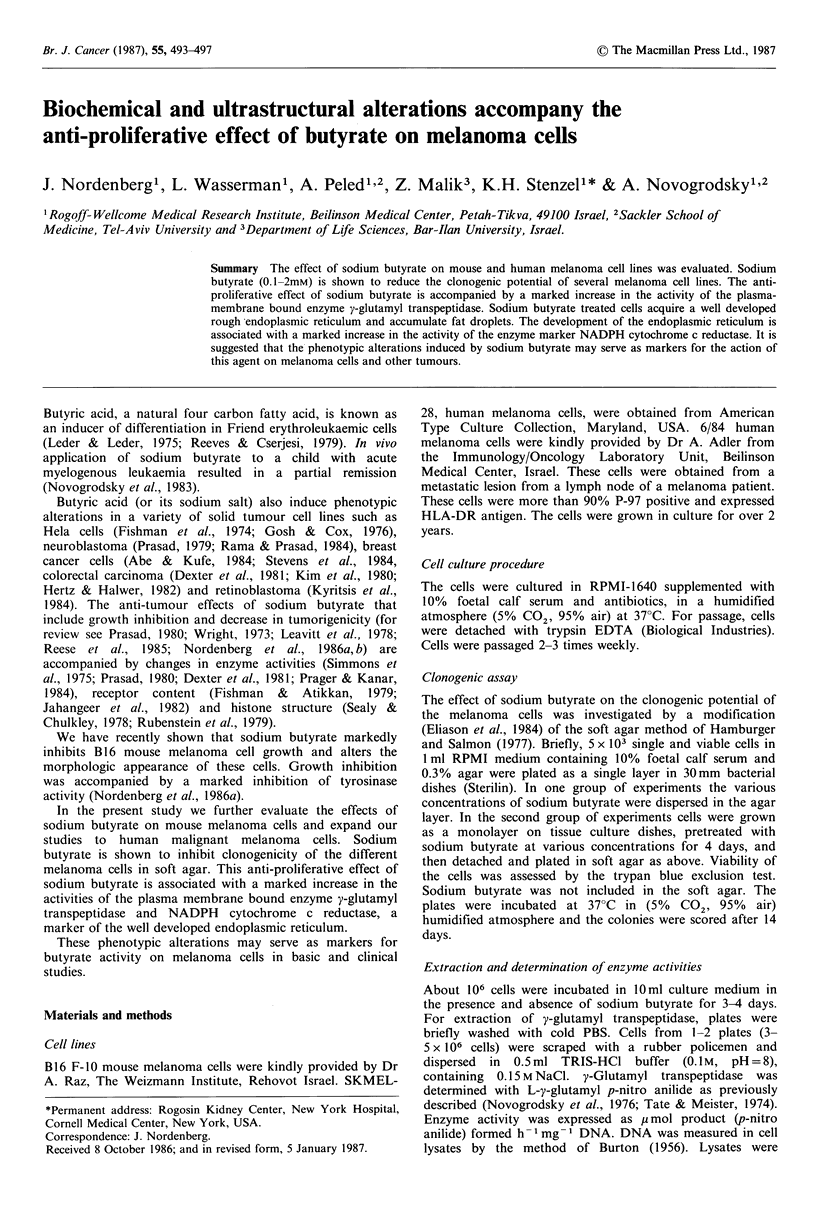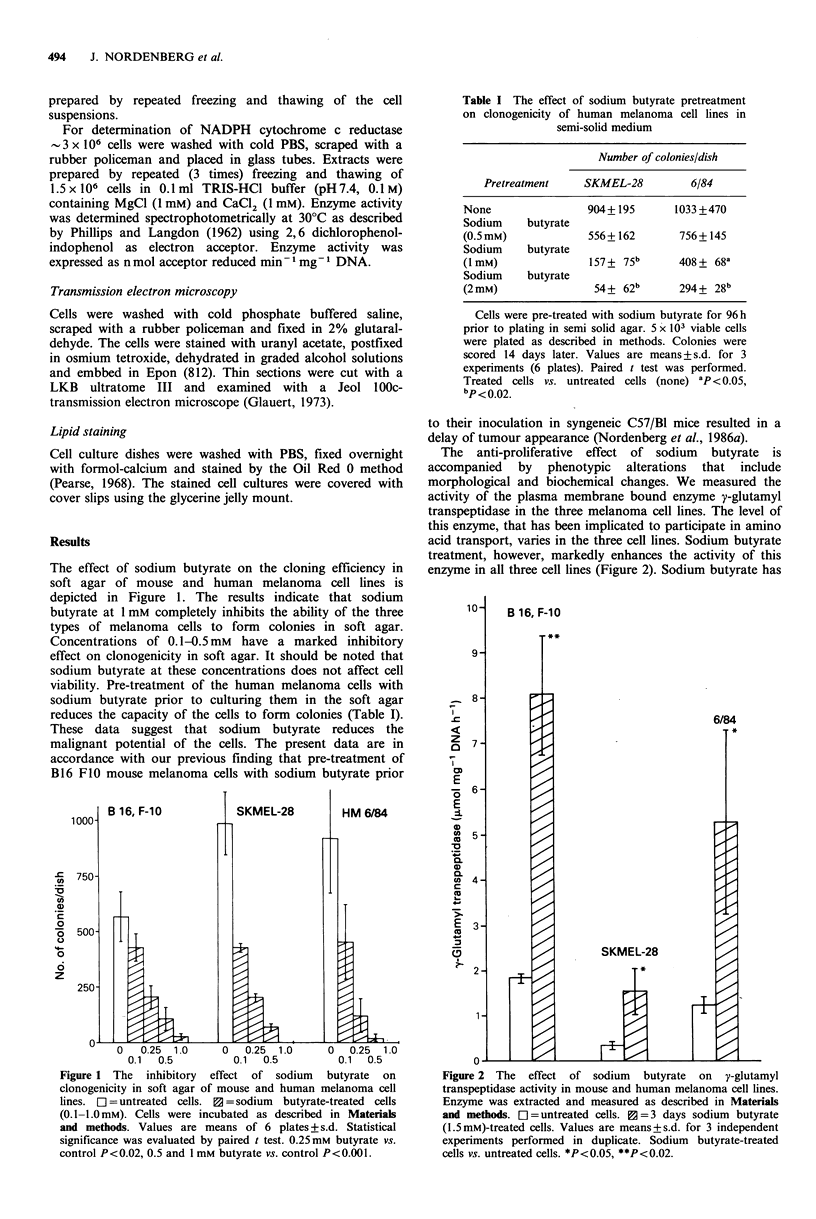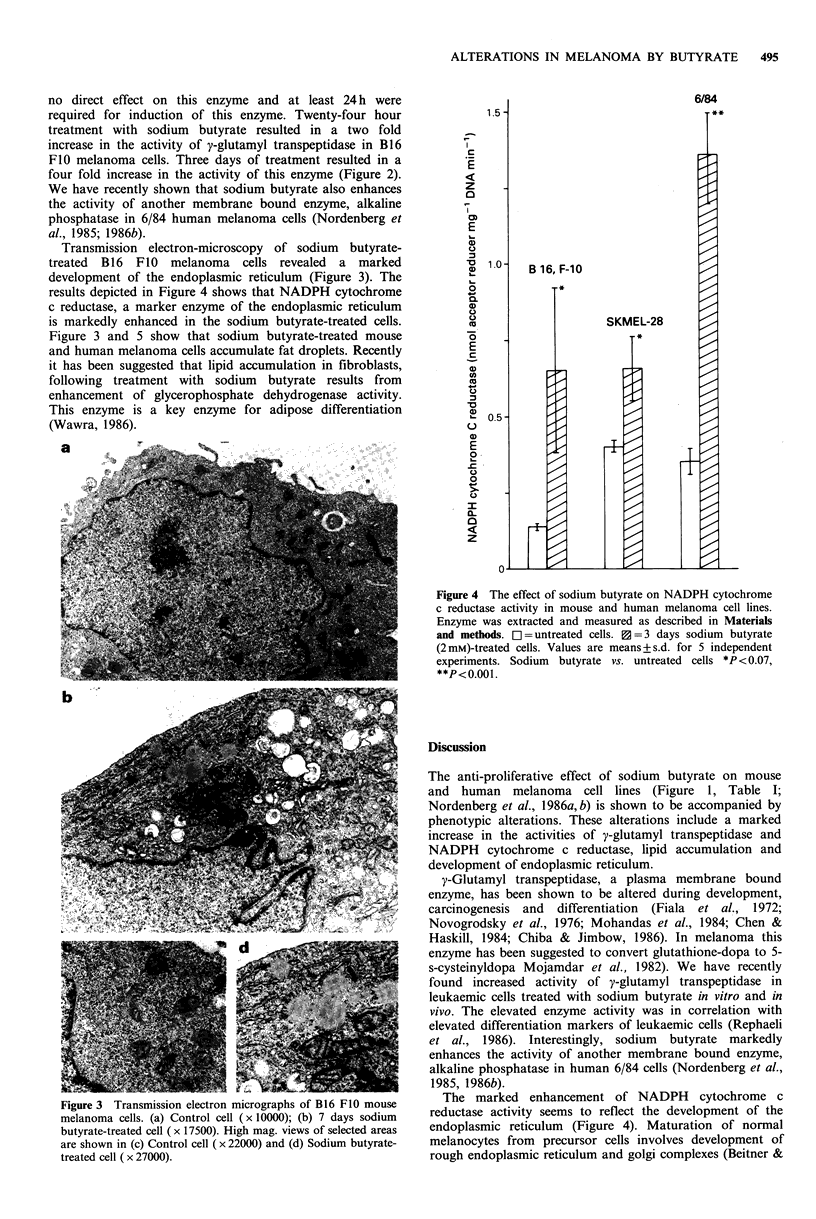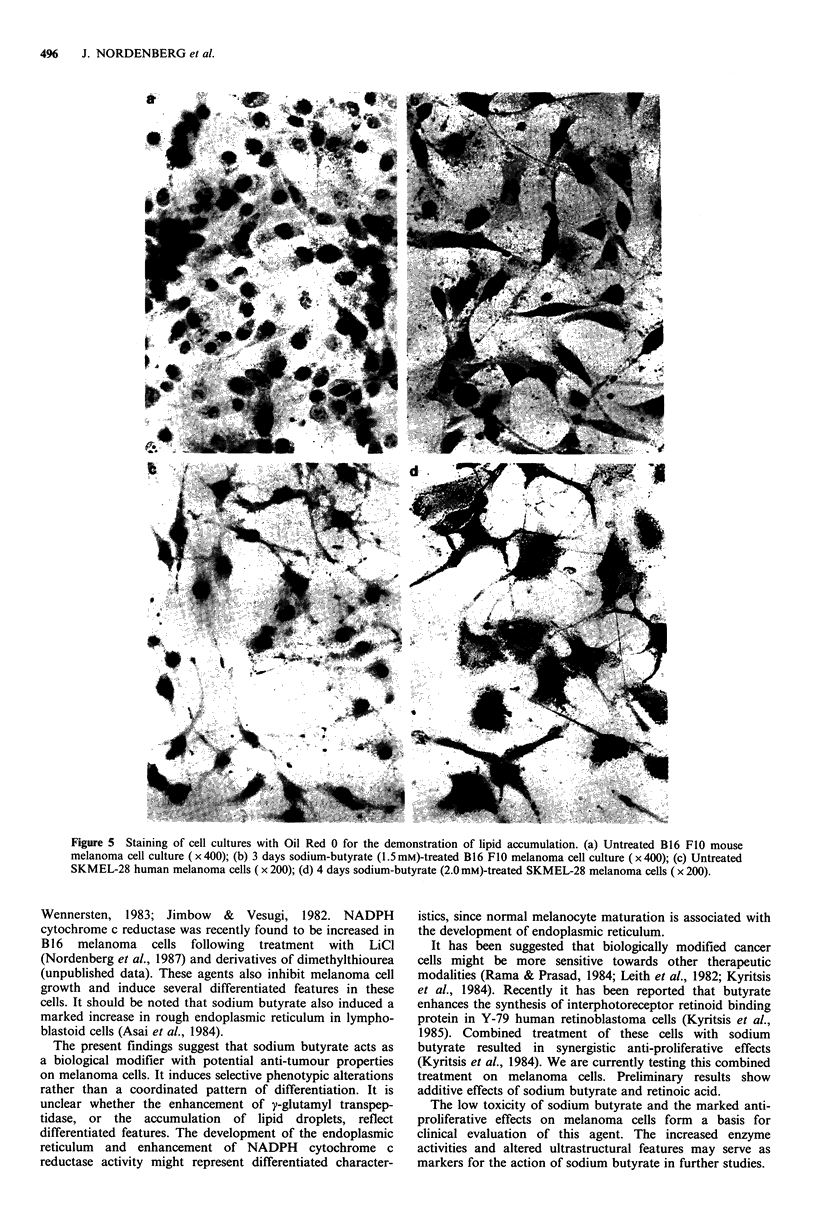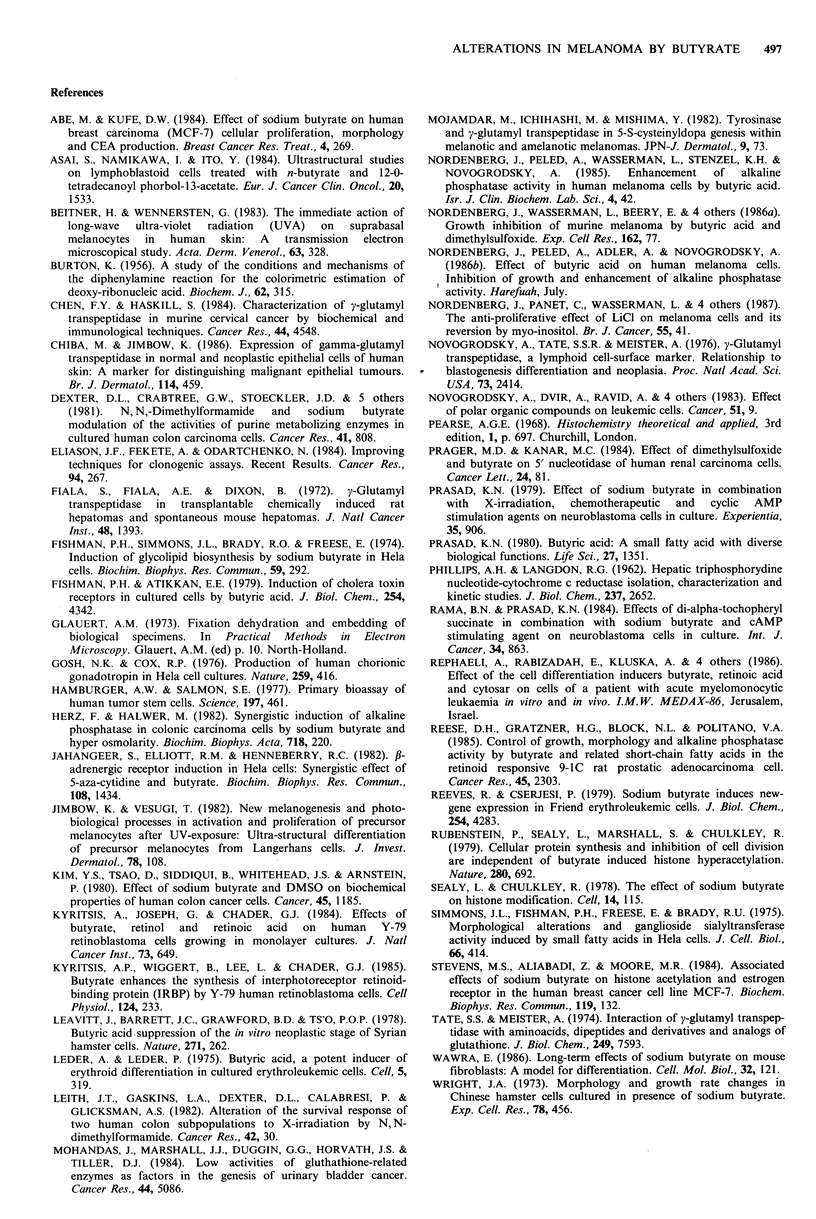# Biochemical and ultrastructural alterations accompany the anti-proliferative effect of butyrate on melanoma cells.

**DOI:** 10.1038/bjc.1987.100

**Published:** 1987-05

**Authors:** J. Nordenberg, L. Wasserman, A. Peled, Z. Malik, K. H. Stenzel, A. Novogrodsky

## Abstract

**Images:**


*Br. J. Cancer* (1987), **55**, 493–497
© The Macmillan Press Ltd., 1987

### Biochemical and ultrastructural alterations accompany the anti-proliferative effect of butyrate on melanoma cells

J. Nordenberg[1], L. Wasserman[1], A. Peled[1,2], Z. Malik[3], K.H. Stenzel[1]* & A. Novogrodsky[1,2]

[1]*Rogoff-Wellcome Medical Research Institute, Beilinson Medical Center, Petah-Tikva, 49100 Israel,* [2]*Sackler School of Medicine, Tel-Aviv University and* [3]*Department of Life Sciences, Bar-Ilan University, Israel.*

**Summary** The effect of sodium butyrate on mouse and human melanoma cell lines was evaluated. Sodium butyrate (0.1–2mM) is shown to reduce the clonogenic potential of several melanoma cell lines. The anti-proliferative effect of sodium butyrate is accompanied by a marked increase in the activity of the plasma-membrane bound enzyme γ-glutamyl transpeptidase. Sodium butyrate treated cells acquire a well developed rough endoplasmic reticulum and accumulate fat droplets. The development of the endoplasmic reticulum is associated with a marked increase in the activity of the enzyme marker NADPH cytochrome c reductase. It is suggested that the phenotypic alterations induced by sodium butyrate may serve as markers for the action of this agent on melanoma cells and other tumours.

Butyric acid, a natural four carbon fatty acid, is known as an inducer of differentiation in Friend erythroleukaemic cells (Leder & Leder, 1975; Reeves & Cserjesi, 1979). *In vivo* application of sodium butyrate to a child with acute myelogenous leukaemia resulted in a partial remission (Novogrodsky *et al.*, 1983).

Butyric acid (or its sodium salt) also induce phenotypic alterations in a variety of solid tumour cell lines such as Hela cells (Fishman *et al.*, 1974; Gosh & Cox, 1976), neuroblastoma (Prasad, 1979; Rama & Prasad, 1984), breast cancer cells (Abe & Kufe, 1984; Stevens *et al.*, 1984, colorectal carcinoma (Dexter *et al.*, 1981; Kim *et al.*, 1980; Hertz & Halwer, 1982) and retinoblastoma (Kyritsis *et al.*, 1984). The anti-tumour effects of sodium butyrate that include growth inhibition and decrease in tumorigenicity (for review see Prasad, 1980; Wright, 1973; Leavitt *et al.*, 1978; Reese *et al.*, 1985; Nordenberg *et al.*, 1986*a*,*b*) are accompanied by changes in enzyme activities (Simmons *et al.*, 1975; Prasad, 1980; Dexter *et al.*, 1981; Prager & Kanar, 1984), receptor content (Fishman & Atikkan, 1979; Jahangeer *et al.*, 1982) and histone structure (Sealy & Chulkley, 1978; Rubenstein *et al.*, 1979).

We have recently shown that sodium butyrate markedly inhibits B16 mouse melanoma cell growth and alters the morphologic appearance of these cells. Growth inhibition was accompanied by a marked inhibition of tyrosinase activity (Nordenberg *et al.*, 1986*a*).

In the present study we further evaluate the effects of sodium butyrate on mouse melanoma cells and expand our studies to human malignant melanoma cells. Sodium butyrate is shown to inhibit clonogenicity of the different melanoma cells in soft agar. This anti-proliferative effect of sodium butyrate is associated with a marked increase in the activities of the plasma membrane bound enzyme γ-glutamyl transpeptidase and NADPH cytochrome c reductase, a marker of the well developed endoplasmic reticulum.

These phenotypic alterations may serve as markers for butyrate activity on melanoma cells in basic and clinical studies.

### Materials and methods

### *Cell lines*

B16 F-10 mouse melanoma cells were kindly provided by Dr A. Raz, The Weizmann Institute, Rehovot Israel. SKMEL-28, human melanoma cells, were obtained from American Type Culture Collection, Maryland, USA. 6/84 human melanoma cells were kindly provided by Dr A. Adler from the Immunology/Oncology Laboratory Unit, Beilinson Medical Center, Israel. These cells were obtained from a metastatic lesion from a lymph node of a melanoma patient. These cells were more than 90% P-97 positive and expressed HLA-DR antigen. The cells were grown in culture for over 2 years.

### *Cell culture procedure*

The cells were cultured in RPMI-1640 supplemented with 10% foetal calf serum and antibiotics, in a humidified atmosphere (5% $CO_2$, 95% air) at 37°C. For passage, cells were detached with trypsin EDTA (Biological Industries). Cells were passaged 2–3 times weekly.

### *Clonogenic assay*

The effect of sodium butyrate on the clonogenic potential of the melanoma cells was investigated by a modification (Eliason *et al.*, 1984) of the soft agar method of Hamburger and Salmon (1977). Briefly, $5\times10^3$ single and viable cells in 1 ml RPMI medium containing 10% foetal calf serum and 0.3% agar were plated as a single layer in 30 mm bacterial dishes (Sterilin). In one group of experiments the various concentrations of sodium butyrate were dispersed in the agar layer. In the second group of experiments cells were grown as a monolayer on tissue culture dishes, pretreated with sodium butyrate at various concentrations for 4 days, and then detached and plated in soft agar as above. Viability of the cells was assessed by the trypan blue exclusion test. Sodium butyrate was not included in the soft agar. The plates were incubated at 37°C in (5% $CO_2$, 95% air) humidified atmosphere and the colonies were scored after 14 days.

### *Extraction and determination of enzyme activities*

About $10^6$ cells were incubated in 10 ml culture medium in the presence and absence of sodium butyrate for 3–4 days. For extraction of γ-glutamyl transpeptidase, plates were briefly washed with cold PBS. Cells from 1–2 plates (3–$5\times10^6$ cells) were scraped with a rubber policemen and dispersed in 0.5 ml TRIS-HCl buffer (0.1M, pH = 8), containing 0.15 M NaCl. γ-Glutamyl transpeptidase was determined with L-γ-glutamyl *p*-nitro anilide as previously described (Novogrodsky *et al.*, 1976; Tate & Meister, 1974). Enzyme activity was expressed as μmol product (*p*-nitro anilide) formed $h^{-1}mg^{-1}$ DNA. DNA was measured in cell lysates by the method of Burton (1956). Lysates were

*Permanent address: Rogosin Kidney Center, New York Hospital, Cornell Medical Center, New York, USA.
Correspondence: J. Nordenberg.
Received 8 October 1986; and in revised form, 5 January 1987.

prepared by repeated freezing and thawing of the cell suspensions.

For determination of NADPH cytochrome c reductase $\sim 3 \times 10^6$ cells were washed with cold PBS, scraped with a rubber policeman and placed in glass tubes. Extracts were prepared by repeated (3 times) freezing and thawing of $1.5 \times 10^6$ cells in 0.1 ml TRIS-HCl buffer (pH 7.4, 0.1 M) containing MgCl (1 mM) and $CaCl_2$ (1 mM). Enzyme activity was determined spectrophotometrically at 30°C as described by Phillips and Langdon (1962) using 2,6 dichlorophenol-indophenol as electron acceptor. Enzyme activity was expressed as n mol acceptor reduced $min^{-1}$ $mg^{-1}$ DNA.

*Transmission electron microscopy*

Cells were washed with cold phosphate buffered saline, scraped with a rubber policeman and fixed in 2% glutaraldehyde. The cells were stained with uranyl acetate, postfixed in osmium tetroxide, dehydrated in graded alcohol solutions and embbed in Epon (812). Thin sections were cut with a LKB ultratome III and examined with a Jeol 100c-transmission electron microscope (Glauert, 1973).

*Lipid staining*

Cell culture dishes were washed with PBS, fixed overnight with formol-calcium and stained by the Oil Red 0 method (Pearse, 1968). The stained cell cultures were covered with cover slips using the glycerine jelly mount.

### Results

The effect of sodium butyrate on the cloning efficiency in soft agar of mouse and human melanoma cell lines is depicted in Figure 1. The results indicate that sodium butyrate at 1 mM completely inhibits the ability of the three types of melanoma cells to form colonies in soft agar. Concentrations of 0.1–0.5 mM have a marked inhibitory effect on clonogenicity in soft agar. It should be noted that sodium butyrate at these concentrations does not affect cell viability. Pre-treatment of the human melanoma cells with sodium butyrate prior to culturing them in the soft agar reduces the capacity of the cells to form colonies (Table I). These data suggest that sodium butyrate reduces the malignant potential of the cells. The present data are in accordance with our previous finding that pre-treatment of B16 F10 mouse melanoma cells with sodium butyrate prior to their inoculation in syngeneic C57/Bl mice resulted in a delay of tumour appearance (Nordenberg *et al.*, 1986*a*).

**Table I** The effect of sodium butyrate pretreatment on clonogenicity of human melanoma cell lines in semi-solid medium

| *Pretreatment* | *Number of colonies/dish* SKMEL-28 | 6/84 |
|---|---|---|
| None | 904±195 | 1033±470 |
| Sodium butyrate (0.5 mM) | 556±162 | 756±145 |
| Sodium butyrate (1 mM) | 157± 75[b] | 408± 68[a] |
| Sodium butyrate (2 mM) | 54± 62[b] | 294± 28[b] |

Cells were pre-treated with sodium butyrate for 96 h prior to plating in semi solid agar. $5 \times 10^3$ viable cells were plated as described in methods. Colonies were scored 14 days later. Values are means±s.d. for 3 experiments (6 plates). Paired *t* test was performed. Treated cells *vs.* untreated cells (none) [a]$P<0.05$, [b]$P<0.02$.

The anti-proliferative effect of sodium butyrate is accompanied by phenotypic alterations that include morphological and biochemical changes. We measured the activity of the plasma membrane bound enzyme γ-glutamyl transpeptidase in the three melanoma cell lines. The level of this enzyme, that has been implicated to participate in amino acid transport, varies in the three cell lines. Sodium butyrate treatment, however, markedly enhances the activity of this enzyme in all three cell lines (Figure 2). Sodium butyrate has

**Figure 1** The inhibitory effect of sodium butyrate on clonogenicity in soft agar of mouse and human melanoma cell lines. □ = untreated cells. ▨ = sodium butyrate-treated cells (0.1–1.0 mM). Cells were incubated as described in **Materials and methods**. Values are means of 6 plates±s.d. Statistical significance was evaluated by paired *t* test. 0.25 mM butyrate *vs.* control $P<0.02$, 0.5 and 1 mM butyrate *vs.* control $P<0.001$.

**Figure 2** The effect of sodium butyrate on γ-glutamyl transpeptidase activity in mouse and human melanoma cell lines. Enzyme was extracted and measured as described in **Materials and methods**. □ = untreated cells. ▨ = 3 days sodium butyrate (1.5 mM)-treated cells. Values are means±s.d. for 3 independent experiments performed in duplicate. Sodium butyrate-treated cells *vs.* untreated cells. *$P<0.05$, **$P<0.02$.

no direct effect on this enzyme and at least 24h were required for induction of this enzyme. Twenty-four hour treatment with sodium butyrate resulted in a two fold increase in the activity of γ-glutamyl transpeptidase in B16 F10 melanoma cells. Three days of treatment resulted in a four fold increase in the activity of this enzyme (Figure 2). We have recently shown that sodium butyrate also enhances the activity of another membrane bound enzyme, alkaline phosphatase in 6/84 human melanoma cells (Nordenberg *et al.*, 1985; 1986*b*).

Transmission electron-microscopy of sodium butyrate-treated B16 F10 melanoma cells revealed a marked development of the endoplasmic reticulum (Figure 3). The results depicted in Figure 4 shows that NADPH cytochrome c reductase, a marker enzyme of the endoplasmic reticulum is markedly enhanced in the sodium butyrate-treated cells. Figure 3 and 5 show that sodium butyrate-treated mouse and human melanoma cells accumulate fat droplets. Recently it has been suggested that lipid accumulation in fibroblasts, following treatment with sodium butyrate results from enhancement of glycerophosphate dehydrogenase activity. This enzyme is a key enzyme for adipose differentiation (Wawra, 1986).

**Figure 3** Transmission electron micrographs of B16 F10 mouse melanoma cells. (a) Control cell (×10000); (b) 7 days sodium butyrate-treated cell (×17500). High mag. views of selected areas are shown in (c) Control cell (×22000) and (d) Sodium butyrate-treated cell (×27000).

**Figure 4** The effect of sodium butyrate on NADPH cytochrome c reductase activity in mouse and human melanoma cell lines. Enzyme was extracted and measured as described in **Materials and methods**. □ = untreated cells. ▨ = 3 days sodium butyrate (2 mM)-treated cells. Values are means ± s.d. for 5 independent experiments. Sodium butyrate *vs.* untreated cells $^*P<0.07$, $^{**}P<0.001$.

### Discussion

The anti-proliferative effect of sodium butyrate on mouse and human melanoma cell lines (Figure 1, Table I; Nordenberg *et al.*, 1986*a*, *b*) is shown to be accompanied by phenotypic alterations. These alterations include a marked increase in the activities of γ-glutamyl transpeptidase and NADPH cytochrome c reductase, lipid accumulation and development of endoplasmic reticulum.

γ-Glutamyl transpeptidase, a plasma membrane bound enzyme, has been shown to be altered during development, carcinogenesis and differentiation (Fiala *et al.*, 1972; Novogrodsky *et al.*, 1976; Mohandas *et al.*, 1984; Chen & Haskill, 1984; Chiba & Jimbow, 1986). In melanoma this enzyme has been suggested to convert glutathione-dopa to 5-s-cysteinyldopa Mojamdar *et al.*, 1982). We have recently found increased activity of γ-glutamyl transpeptidase in leukaemic cells treated with sodium butyrate *in vitro* and *in vivo*. The elevated enzyme activity was in correlation with elevated differentiation markers of leukaemic cells (Rephaeli *et al.*, 1986). Interestingly, sodium butyrate markedly enhances the activity of another membrane bound enzyme, alkaline phosphatase in human 6/84 cells (Nordenberg *et al.*, 1985, 1986*b*).

The marked enhancement of NADPH cytochrome c reductase activity seems to reflect the development of the endoplasmic reticulum (Figure 4). Maturation of normal melanocytes from precursor cells involves development of rough endoplasmic reticulum and golgi complexes (Beitner &

**Figure 5** Staining of cell cultures with Oil Red 0 for the demonstration of lipid accumulation. (a) Untreated B16 F10 mouse melanoma cell culture (×400); (b) 3 days sodium-butyrate (1.5 mM)-treated B16 F10 melanoma cell culture (×400); (c) Untreated SKMEL-28 human melanoma cells (×200); (d) 4 days sodium-butyrate (2.0 mM)-treated SKMEL-28 melanoma cells (×200).

Wennersten, 1983; Jimbow & Vesugi, 1982. NADPH cytochrome c reductase was recently found to be increased in B16 melanoma cells following treatment with LiCl (Nordenberg *et al.*, 1987) and derivatives of dimethylthiourea (unpublished data). These agents also inhibit melanoma cell growth and induce several differentiated features in these cells. It should be noted that sodium butyrate also induced a marked increase in rough endoplasmic reticulum in lymphoblastoid cells (Asai *et al.*, 1984).

The present findings suggest that sodium butyrate acts as a biological modifier with potential anti-tumour properties on melanoma cells. It induces selective phenotypic alterations rather than a coordinated pattern of differentiation. It is unclear whether the enhancement of γ-glutamyl transpeptidase, or the accumulation of lipid droplets, reflect differentiated features. The development of the endoplasmic reticulum and enhancement of NADPH cytochrome c reductase activity might represent differentiated characteristics, since normal melanocyte maturation is associated with the development of endoplasmic reticulum.

It has been suggested that biologically modified cancer cells might be more sensitive towards other therapeutic modalities (Rama & Prasad, 1984; Leith *et al.*, 1982; Kyritsis *et al.*, 1984). Recently it has been reported that butyrate enhances the synthesis of interphotoreceptor retinoid binding protein in Y-79 human retinoblastoma cells (Kyritsis *et al.*, 1985). Combined treatment of these cells with sodium butyrate resulted in synergistic anti-proliferative effects (Kyritsis *et al.*, 1984). We are currently testing this combined treatment on melanoma cells. Preliminary results show additive effects of sodium butyrate and retinoic acid.

The low toxicity of sodium butyrate and the marked anti-proliferative effects on melanoma cells form a basis for clinical evaluation of this agent. The increased enzyme activities and altered ultrastructural features may serve as markers for the action of sodium butyrate in further studies.

### References

ABE, M. & KUFE, D.W. (1984). Effect of sodium butyrate on human breast carcinoma (MCF-7) cellular proliferation, morphology and CEA production. *Breast Cancer Res. Treat.*, **4**, 269.

ASAI, S., NAMIKAWA, I. & ITO, Y. (1984). Ultrastructural studies on lymphoblastoid cells treated with *n*-butyrate and 12-0-tetradecanoyl phorbol-13-acetate. *Eur. J. Cancer Clin. Oncol.*, **20**, 1533.

BEITNER, H. & WENNERSTEN, G. (1983). The immediate action of long-wave ultra-violet radiation (UVA) on suprabasal melanocytes in human skin: A transmission electron microscopical study. *Acta. Derm. Venerol.*, **63**, 328.

BURTON, K. (1956). A study of the conditions and mechanisms of the diphenylamine reaction for the colorimetric estimation of deoxy-ribonucleic acid. *Biochem. J.*, **62**, 315.

CHEN, F.Y. & HASKILL, S. (1984). Characterization of γ-glutamyl transpeptidase in murine cervical cancer by biochemical and immunological techniques. *Cancer Res.*, **44**, 4548.

CHIBA, M. & JIMBOW, K. (1986). Expression of gamma-glutamyl transpeptidase in normal and neoplastic epithelial cells of human skin: A marker for distinguishing malignant epithelial tumours. *Br. J. Dermatol.*, **114**, 459.

DEXTER, D.L., CRABTREE, G.W., STOECKLER, J.D. & 5 others (1981). N, N,-Dimethylformamide and sodium butyrate modulation of the activities of purine metabolizing enzymes in cultured human colon carcinoma cells. *Cancer Res.*, **41**, 808.

ELIASON, J.F., FEKETE, A. & ODARTCHENKO, N. (1984). Improving techniques for clonogenic assays. Recent Results. *Cancer Res.*, **94**, 267.

FIALA, S., FIALA, A.E. & DIXON, B. (1972). γ-Glutamyl transpeptidase in transplantable chemically induced rat hepatomas and spontaneous mouse hepatomas. *J. Natl Cancer Inst.*, **48**, 1393.

FISHMAN, P.H., SIMMONS, J.L., BRADY, R.O. & FREESE, E. (1974). Induction of glycolipid biosynthesis by sodium butyrate in Hela cells. *Biochim. Biophys. Res. Commun.*, **59**, 292.

FISHMAN, P.H. & ATIKKAN, E.E. (1979). Induction of cholera toxin receptors in cultured cells by butyric acid. *J. Biol. Chem.*, **254**, 4342.

GLAUERT, A.M. (1973). Fixation dehydration and embedding of biological specimens. In *Practical Methods in Electron Microscopy*. Glauert, A.M. (ed) p. 10. North-Holland.

GOSH, N.K. & COX, R.P. (1976). Production of human chorionic gonadotropin in Hela cell cultures. *Nature*, **259**, 416.

HAMBURGER, A.W. & SALMON, S.E. (1977). Primary bioassay of human tumor stem cells. *Science*, **197**, 461.

HERZ, F. & HALWER, M. (1982). Synergistic induction of alkaline phosphatase in colonic carcinoma cells by sodium butyrate and hyper osmolarity. *Biochim. Biophys. Acta*, **718**, 220.

JAHANGEER, S., ELLIOTT, R.M. & HENNEBERRY, R.C. (1982). β-adrenergic receptor induction in Hela cells: Synergistic effect of 5-aza-cytidine and butyrate. *Biochim. Biophys. Res. Commun.*, **108**, 1434.

JIMBOW, K. & VESUGI, T. (1982). New melanogenesis and photo-biological processes in activation and proliferation of precursor melanocytes after UV-exposure: Ultra-structural differentiation of precursor melanocytes from Langerhans cells. *J. Invest. Dermatol.*, **78**, 108.

KIM, Y.S., TSAO, D., SIDDIQUI, B., WHITEHEAD, J.S. & ARNSTEIN, P. (1980). Effect of sodium butyrate and DMSO on biochemical properties of human colon cancer cells. *Cancer*, **45**, 1185.

KYRITSIS, A., JOSEPH, G. & CHADER, G.J. (1984). Effects of butyrate, retinol and retinoic acid on human Y-79 retinoblastoma cells growing in monolayer cultures. *J. Natl Cancer Inst.*, **73**, 649.

KYRITSIS, A.P., WIGGERT, B., LEE, L. & CHADER, G.J. (1985). Butyrate enhances the synthesis of interphotoreceptor retinoid-binding protein (IRBP) by Y-79 human retinoblastoma cells. *Cell Physiol.*, **124**, 233.

LEAVITT, J., BARRETT, J.C., GRAWFORD, B.D. & TS'O, P.O.P. (1978). Butyric acid suppression of the *in vitro* neoplastic stage of Syrian hamster cells. *Nature*, **271**, 262.

LEDER, A. & LEDER, P. (1975). Butyric acid, a potent inducer of erythroid differentiation in cultured erythroleukemic cells. *Cell*, **5**, 319.

LEITH, J.T., GASKINS, L.A., DEXTER, D.L., CALABRESI, P. & GLICKSMAN, A.S. (1982). Alteration of the survival response of two human colon subpopulations to X-irradiation by N,N-dimethylformamide. *Cancer Res.*, **42**, 30.

MOHANDAS, J., MARSHALL, J.J., DUGGIN, G.G., HORVATH, J.S. & TILLER, D.J. (1984). Low activities of gluthathione-related enzymes as factors in the genesis of urinary bladder cancer. *Cancer Res.*, **44**, 5086.

MOJAMDAR, M., ICHIHASHI, M. & MISHIMA, Y. (1982). Tyrosinase and γ-glutamyl transpeptidase in 5-S-cysteinyldopa genesis within melanotic and amelanotic melanomas. JPN-*J. Dermatol.*, **9**, 73.

NORDENBERG, J., PELED, A., WASSERMAN, L., STENZEL, K.H. & NOVOGRODSKY, A. (1985). Enhancement of alkaline phosphatase activity in human melanoma cells by butyric acid. *Isr. J. Clin. Biochem. Lab. Sci.*, **4**, 42.

NORDENBERG, J., WASSERMAN, L., BEERY, E. & 4 others (1986*a*). Growth inhibition of murine melanoma by butyric acid and dimethylsulfoxide. *Exp. Cell Res.*, **162**, 77.

NORDENBERG, J., PELED, A., ADLER, A. & NOVOGRODSKY, A. (1986*b*). Effect of butyric acid on human melanoma cells. Inhibition of growth and enhancement of alkaline phosphatase activity. *Harefuah*, July.

NORDENBERG, J., PANET, C., WASSERMAN, L. & 4 others (1987). The anti-proliferative effect of LiCl on melanoma cells and its reversion by myo-inositol. *Br. J. Cancer*, **55**, 41.

NOVOGRODSKY, A., TATE, S.S.R. & MEISTER, A. (1976). γ-Glutamyl transpeptidase, a lymphoid cell-surface marker. Relationship to blastogenesis differentiation and neoplasia. *Proc. Natl Acad. Sci. USA*, **73**, 2414.

NOVOGRODSKY, A., DVIR, A., RAVID, A. & 4 others (1983). Effect of polar organic compounds on leukemic cells. *Cancer*, **51**, 9.

PEARSE, A.G.E. (1968). *Histochemistry theoretical and applied*, 3rd edition, **1**, p. 697. Churchill, London.

PRAGER, M.D. & KANAR, M.C. (1984). Effect of dimethylsulfoxide and butyrate on 5′ nucleotidase of human renal carcinoma cells. *Cancer Lett.*, **24**, 81.

PRASAD, K.N. (1979). Effect of sodium butyrate in combination with X-irradiation, chemotherapeutic and cyclic AMP stimulation agents on neuroblastoma cells in culture. *Experientia*, **35**, 906.

PRASAD, K.N. (1980). Butyric acid: A small fatty acid with diverse biological functions. *Life Sci.*, **27**, 1351.

PHILLIPS, A.H. & LANGDON, R.G. (1962). Hepatic triphosphorydine nucleotide-cytochrome c reductase isolation, characterization and kinetic studies. *J. Biol. Chem.*, **237**, 2652.

RAMA, B.N. & PRASAD, K.N. (1984). Effects of di-alpha-tochopheryl succinate in combination with sodium butyrate and cAMP stimulating agent on neuroblastoma cells in culture. *Int. J. Cancer*, **34**, 863.

REPHAELI, A., RABIZADAH, E., KLUSKA, A. & 4 others (1986). Effect of the cell differentiation inducers butyrate, retinoic acid and cytosar on cells of a patient with acute myelomonocytic leukaemia *in vitro* and *in vivo*. *I.M.W. MEDAX-86*, Jerusalem, Israel.

REESE, D.H., GRATZNER, H.G., BLOCK, N.L. & POLITANO, V.A. (1985). Control of growth, morphology and alkaline phosphatase activity by butyrate and related short-chain fatty acids in the retinoid responsive 9-1C rat prostatic adenocarcinoma cell. *Cancer Res.*, **45**, 2303.

REEVES, R. & CSERJESI, P. (1979). Sodium butyrate induces new-gene expression in Friend erythroleukemic cells. *J. Biol. Chem.*, **254**, 4283.

RUBENSTEIN, P., SEALY, L., MARSHALL, S. & CHULKLEY, R. (1979). Cellular protein synthesis and inhibition of cell division are independent of butyrate induced histone hyperacetylation. *Nature*, **280**, 692.

SEALY, L. & CHULKLEY, R. (1978). The effect of sodium butyrate on histone modification. *Cell*, **14**, 115.

SIMMONS, J.L., FISHMAN, P.H., FREESE, E. & BRADY, R.U. (1975). Morphological alterations and ganglioside sialyltransferase activity induced by small fatty acids in Hela cells. *J. Cell. Biol.*, **66**, 414.

STEVENS, M.S., ALIABADI, Z. & MOORE, M.R. (1984). Associated effects of sodium butyrate on histone acetylation and estrogen receptor in the human breast cancer cell line MCF-7. *Biochem. Biophys. Res. Commun.*, **119**, 132.

TATE, S.S. & MEISTER, A. (1974). Interaction of γ-glutamyl transpeptidase with aminoacids, dipeptides and derivatives and analogs of glutathione. *J. Biol. Chem.*, **249**, 7593.

WAWRA, E. (1986). Long-term effects of sodium butyrate on mouse fibroblasts: A model for differentiation. *Cell. Mol. Biol.*, **32**, 121.

WRIGHT, J.A. (1973). Morphology and growth rate changes in Chinese hamster cells cultured in presence of sodium butyrate. *Exp. Cell. Res.*, **78**, 456.